# Emergent Strain of Human Adenovirus Endemic in Iowa

**DOI:** 10.3201/eid1101.040490

**Published:** 2005-01

**Authors:** Gregory C. Gray, Sharon F. Setterquist, Sandra J. Jirsa, Lucy E. DesJardin, Dean D. Erdman

**Affiliations:** * University of Iowa College of Public Health, Iowa City, Iowa, USA; † University of Iowa Hygienic Laboratory, Iowa City, Iowa, USA; ‡ Centers for Disease Control and Prevention, Atlanta, Georgia, USA

**Keywords:** Adenoviridae, adenoviruses, adenovirus infections, epidemiology, vaccines, respiratory infections, respiratory tract infections, dispatch

## Abstract

We evaluated 76 adenovirus type 7 (Ad7) isolates collected in Iowa from 1992 to 2002 and found that genome type Ad7d2 became increasingly prevalent. By 2002, it had supplanted all other Ad7 genome types. The association of Ad7d2 with severe illness and death calls for heightened public health concern.

Human adenoviruses are the cause of a wide spectrum acute and chronic diseases. The associations of adenovirus with keratoconjunctivitis, upper respiratory tract infections, pneumonia, gastroenteritis, cystitis, and encephalitis have long been recognized. Recently, molecular methods have shown adenoviruses to be associated with bronchopulmonary dysplasia (*1*), chronic obstructive pulmonary disease (*2*), and mycocarditis (*3*). Adenovirus infections cause severe illness and death in immunocompromised persons, particularly bone marrow transplant recipients (*4*–*6*).

In 2002, Erdman et al. (*7*) reported that 2 emergent genome types of adenovirus type 7 (Ad7) had recently been detected in North American populations. From restriction enzyme studies of 166 archived specimens, the available data suggested that Ad7d2 and Ad7h first appeared in North America in 1993 and 1998, respectively. Both genome types had been associated with epidemics, severe illness, and deaths in populations outside the United States. Since Ad7d2 has been associated with 3 military and 3 civilian epidemics and at least 19 deaths in the United States since 1993, the 2002 report voiced concern regarding a shift in the prevalence of U.S. adenovirus strains and the need to increase surveillance for adenoviral disease. We present a retrospective study of Ad7 isolates in Iowa.

## The Study

By using a previously described DNA restriction analysis procedure (*7*), we studied 76 archived adenovirus isolates collected among influenzalike-illness surveillance sites across Iowa from 1992 to 2002.

Among the 76 isolates, 40 (53%) were Ad7d2, and 6 (8%) were Ad7h (Figure). The first Ad7d2 specimen was isolated in March 1994 from a child living in south-central Iowa. The first Ad7h specimen was isolated in November 1993 from a child living in north-central Iowa. The latter specimen is the earliest Ad7h detected in North America.

**Figure:**
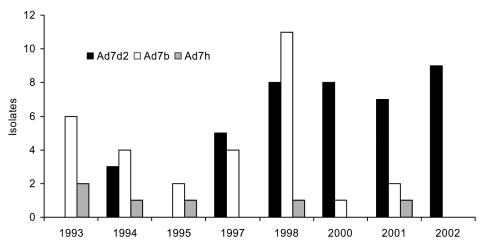
Number of adenovirus (Ad) isolates collected in Iowa during influenzalike-illness surveillance by genome type and year.

Ad7d2 caused illness among patients in Iowa ranging in age from 3 months to 49 years. Of the patients, 75% were male (cause of overrepresentation is unknown). Although the clinical details are sparse, a number of patients were thought to have influenza or were diagnosed with respiratory distress syndrome. At least 4 children from an Ad7d2 October 2000 epidemic at a long-term care facility in Des Moines, Iowa, died. Ad7d2 isolates were obtained from 12 different sites in Iowa. Ad7h was detected in 4 Iowa counties.

Beginning in 1994, Ad7d2 became increasingly more prevalent across Iowa, displacing Ad7b, the predominant genome type circulating in the United States since the early 1970s (*8*). In 2002, data suggest that Ad7d2 supplanted all other Ad7 genome types (9 of 9 Ad7 isolates were A7d2) (Figure).

## Conclusions

Ad7d2 and Ad7h have only recently been recognized. Ad7d2 was first detected in Israel in 1992; beginning in 1995, it was associated with epidemics of unusually severe respiratory disease with high fevers among children in Japan (*9*,*10*). Ad7h was first detected in South America in 1986; since then it has supplanted the previous most prevalent genome type, Ad7c, in Chile, Uruguay, Argentina, and possibly other countries (*11*). Ad7h has caused pediatric respiratory epidemics, and infected children had longer hospitalizations, had higher temperatures, and required more supplemental oxygen (*12*). In at least 1 study, up to 94% of adenovirus deaths were attributed to Ad7h (*11*).

Whether these strains are truly more virulent or whether they better evade the host’s immune system is a matter for future study. What does seem to be clear is that a simple mutation (Ad7d2) (*9*) or recombination (Ad7h) (*13*) may generate new adenovirus strains that could result in more epidemics and higher death rates. These strains may then quickly migrate to new areas and cause more epidemics.

Current U.S. surveillance for adenovirus is passive and incomplete. The number of immunocompromised patients in the United States is increasing, and they, in addition to young children, may be at increased risk for severe disease from emergent adenovirus strains. Developing molecular typing strategies for emerging Ad strains seems prudent, as does improving local and national surveillance for adenovirus illness. Considering adenovirus to be a potential nosocomial pathogen seems wise, and researchers should seek to identify effective antiviral therapy for outbreak interventions. These actions will help public health officials better understand the changing epidemiology of adenovirus infections. Because of increased adenovirus morbidity (*14*,*15*), the U.S. Department of Defense recently contracted to again produce Ad4 and Ad7 vaccines for military trainees. If civilian populations were identified to be at high risk for serious Ad4 or Ad7 disease, they might also benefit from these vaccines.
